# 24-epibrassinolide-mediated regulation of physiological and biochemical parameters in *Chlamydomonas reinhardtii* for optimized growth and future biotechnological applications

**DOI:** 10.3389/fpls.2026.1852756

**Published:** 2026-06-23

**Authors:** Jan Żeruń, Andrzej Bajguz

**Affiliations:** 1 Doctoral School of Exact and Natural Sciences, University of Bialystok, Bialystok, Poland; 2 Department of Biology and Plant Ecology, Faculty of Biology, University of Bialystok, Bialystok, Poland

**Keywords:** 24-epibrassinolide, brassinosteroids, *Chlamydomonas reinhardtii*, fatty acids, microalgae

## Abstract

Brassinosteroids (BRs) are essential steroidal phytohormones regulating plant growth, metabolism, and stress responses; however, their physiological role in microalgae remains insufficiently characterized. The present study evaluated the concentration-dependent effects of 24-epibrassinolide (24-epiBL) on growth dynamics and metabolic composition in the green microalga *Chlamydomonas reinhardtii*. Cultures were exposed to 24-epiBL at concentrations ranging from 0.1 to 1000 nM, and parameters including cell number, protein and monosaccharide content, fatty acid composition, and photosynthetic pigment levels were assessed over a 17-day cultivation period. Low nanomolar concentrations of 24-epiBL induced a hormetic response, with 1 nM identified as the optimal concentration. This treatment significantly increased cell density, enhanced protein and monosaccharide accumulation, and modified lipid composition toward a higher proportion of unsaturated fatty acids. In addition, 1 nM 24-epiBL stimulated the accumulation of photosynthetic pigments and carotenoids, indicating improved photosynthetic capacity and metabolic performance. In contrast, higher concentrations inhibited growth and altered metabolic profiles, including increased relative levels of saturated and monounsaturated fatty acids, consistent with a shift toward a less favorable physiological state. These findings demonstrate that 24-epiBL regulates growth and metabolic pathways in *C. reinhardtii* in a concentration-dependent manner and support its physiological relevance in microalgae. The results extend current knowledge of BR function beyond higher plants and highlight the potential of BRs as tools for modulating biomass composition and metabolic traits in algal biotechnology.

## 1 Introduction

Algae represent a highly diverse group of photosynthetic organisms encompassing multiple evolutionary lineages. Many algal species possess properties of considerable value for human use, ranging from nutritional applications to industrial processes and environmental remediation (Singh and Saxena, 2015). Despite their high photosynthetic efficiency, microalgae often exhibit limited biomass productivity under large-scale cultivation conditions and are highly sensitive to environmental fluctuations, which constrains their industrial exploitation (Singh and Saxena, 2015; Sharma and Sharma, 2017). Nevertheless, algae remain indispensable in numerous applications, as reflected by the widespread commercial use of *Arthrospira* spp. and *Chlorella* spp. as dietary supplements, as well as the utilization of algal-derived products such as agar in the food, cosmetic, pharmaceutical, and feed industries (Priyadarshani and Rath, 2012).

One microalgal species with particularly high biotechnological potential is *Chlamydomonas reinhardtii*, which has been established as a model organism and a versatile platform for applications such as recombinant protein production and metabolic engineering (Pang et al., 2019; Darwish et al., 2020; Jiji et al., 2024). Despite extensive evidence of its nutritional and biomedical potential, *C. reinhardtii* is not yet produced on a large commercial scale, primarily due to limitations in biomass productivity and cultivation stability (Zhang et al., 2019). As a unicellular microalga, it is highly sensitive to environmental perturbations and contamination, making stable, high-yield cultivation challenging. Consequently, strategies that enhance growth efficiency while improving cellular stress tolerance are required to fully exploit its biotechnological potential. One promising group of compounds that may fulfill this role are brassinosteroids (BRs), a class of steroidal phytohormones known to regulate cell growth, development, and stress responses. In higher plants, BRs promote growth by modulating transcriptional and translational activity and by regulating numerous metabolic pathways (Bajguz, 2000, Bajguz, 2007). In addition, BRs play a crucial role in stress tolerance, as their endogenous levels and signaling activity increase in response to both biotic and abiotic stressors (Żeruń and Bajguz, 2025). BRs have been identified across the plant kingdom, including representatives of multiple algal lineages, underscoring their evolutionary conservation and supporting their physiological relevance in algae (Rehman et al., 2022). Similar to animal steroid hormones, exogenously applied BRs can significantly influence cellular metabolism, with their effects strongly dependent on concentration (Bajguz, 2000, Bajguz, 2007). Numerous studies have demonstrated that BRs exhibit hormetic behavior, stimulating growth and metabolic activity at low concentrations while exerting inhibitory or stress-related effects at higher doses (Bajguz and Czerpak, 1996; Bajguz, 2010). Therefore, the application of exogenous BRs in algal cultures requires careful evaluation to identify concentration ranges that enhance desirable biochemical traits without disrupting cellular homeostasis.

From both physiological and applied perspectives, key parameters determining the value of algal biomass include growth rate, protein, carbohydrate and pigments content, and lipid composition (Becker, 2007; Chu, 2017; Liu et al., 2024). These traits directly influence production efficiency and economic feasibility in industries such as food, feed, biofuel, and pharmaceutical manufacturing. Understanding how BRs modulate these parameters in microalgae is therefore essential for both fundamental research and biotechnological applications.

In this study, we investigated the effects of exogenous 24-epiBL, one of the most biologically active BRs, on the growth dynamics and selected biochemical parameters of *C. reinhardtii*. Similar studies were led for species like *Chlorella vulgaris* (Bajguz, 2010; Bajguz and Piotrowska-Niczyporuk, 2014), *Acutodesmus obliquus* (Talarek-Karwel et al., 2020) or *Scenedesmus quadricauda* (Kozlova et al., 2018) but there has not been such research led on *C. reinhardtii* yet, which is very promising, model species. It is a well-established principle that phytohormones typically exhibit a dose-dependent, biphasic action, where low to moderate concentrations stimulate physiological processes, whereas higher doses often lead to growth inhibition or toxicity (Piotrowska-Niczyporuk and Bajguz, 2014; Nolan et al., 2020). Given this universal mechanism and the striking lack of knowledge regarding BRs effects on microalgae, we hypothesized that moderate concentrations of 24-epiBL would induce a hormetic response, enhancing cell proliferation, protein and monosaccharide accumulation, modifying lipid composition, and increasing the content of photosynthetic pigments and non-enzymatic antioxidant carotenoids. Conversely, supra-optimal concentrations were expected to impair growth and metabolic balance, reflecting a shift toward a less favorable physiological state. By characterizing these dose-dependent responses over time, this study aims to clarify the role of BRs in microalgal physiology and to evaluate their potential for improving metabolic performance and stress tolerance in *C. reinhardtii* under controlled cultivation conditions.

## 2 Materials and methods

### 2.1 Algal strain and culture conditions

The experiments were conducted using a wild type *Chlamydomonas reinhardtii* strain SAG 73.72 (Culture Collection of Algae at Göttingen University, Germany) which was maintained in our institutional laboratory and used for all investigations. Algal cultures were grown in tris-acetate-phosphate (TAP) medium (pH 7.0), prepared according to the supplier’s recommendations and established protocols (Harris, 1989; Andersen, 2005). Prior to experimental treatments, a maternal culture was maintained for two weeks in a phytotron under optimal laboratory conditions for *C. reinhardtii*: temperature 23–25 °C, light intensity 300 µmol m^-2^ s^-1^, and a 12h light/12h dark photoperiod (Harris, 2009). When the culture reached a high cell density, it was harvested by centrifugation, the supernatant was discarded, and the cells were transferred to high-salt medium for synchronization. Synchronization was carried out for three days at 28–30 °C, under a 13 h light/11 h dark photoperiod and light intensity of 500 µmol m^-2^ s^-1^ (Hlavová et al., 2016). After synchronization, cells were centrifuged again and resuspended in fresh TAP medium.

### 2.2 Experimental design and brassinosteroid treatments

Experimental cultures were established in 0.5 L Erlenmeyer flasks containing TAP medium. 24-epibrassinolide (24-epiBL) was applied at final concentrations of 0.1, 1, 10, 100, and 1000 nM. Control cultures were maintained under identical conditions without the addition of BR. 24-epiBL was dissolved in 96% ethanol. The same amount of different stock solutions were added to proper flasks. To the ones with control cultures, the same amount of ethanol, without 24-epiBL was added giving us a final concentration of 0,00456% of ethanol in every flask which is trace quantity. Cultures were grown under the same temperature (23–25 °C), light intensity (300 µmol m^-2^ s^-1^), and photoperiod (12h) conditions as described above. Added algae were in their exponential growth phase after synchronization. The experiment was conducted over a 17-day cultivation period letting algae cultures grow from exponential phase to stationary phase. Samples were collected on days 0, 3, and 5, and subsequently at three-day intervals. The experimental design involved 6 treatment conditions (the control and five concentrations of 24-epiBL). To prevent changes in culture volume from repeated sampling, independent cultures were established for each of the 6 subsequent sampling points (days 3, 5, 8, 11, 14, and 17). Each condition at each specific time point was set up in two independent biological replicates (flasks), resulting in a total of 72 experimental cultures (6 treatments × 6 time points × 2 biological replicates). The whole experiment was performed four times independently, using separate inoculates in the same stage of growth, after synchronization. It should not affect the comparison between experimental repeats since all cultures were established to the same optical density and all of the parameters were calculated as the amount per cell. Baseline measurements (Day 0) were obtained from the initial stock culture prior to treatment allocation. To ensure statistical robustness, samples from each biological replicate were analyzed in duplicate (two technical replicates), yielding four data points per condition. To maintain constant culture volumes and light penetration profiles throughout the experiment, a destructive sampling strategy was employed.

### 2.3 Cell counting and biochemical analyses

Cell number was determined using a Bürker counting chamber. Protein content was quantified using the Bradford method, while monosaccharide content was measured using the Samogyi–Nelson method. Absorbance measurements were performed using a Hitachi U-5100 UV–Vis spectrophotometer at wavelengths of 595 nm (proteins) and 540 nm (monosaccharides), respectively. Protein and monosaccharide contents were normalized to cell number.

Photosynthetic pigments were analyzed using high-performance liquid chromatography (HPLC) with a diode-array detector. Analyses were performed on an Eclipse XDB C_8_ column (150 mm × 4.6 mm). The injection volume was 300 µL, and the flow rate was set to 0.8 mL min^-1^. Absorption spectra of the pigments were continuously recorded in the range of 450–665 nm with 20 nm band width, using a diode-array detector (DAD) For pigment extraction, 10 mL of algal culture was centrifuged, the supernatant discarded, and the pellet transferred to 1.5 mL microcentrifuge tubes and centrifuged again to remove residual medium. Pigments were extracted using 1 mL of 99.9% methanol (HPLC grade), followed by purification using Millipore centrifugal filters with a 0.22µm pore size. Separation was performed according to Zapata et al. (2000) method.

Fatty acids were extracted from dried and weighed algal biomass using a mixture of ethanol, chloroform, and hexane. Following extraction, organic solvents were evaporated in a 70 °C water bath under ultrasonic agitation. The resulting residue was reconstituted in 200 µL of hexane and transferred to chromatographic vials. Fatty acid profiles were determined using gas chromatography coupled with mass spectroscopy (Niemi et al., 2019).

### 2.4 Statistical analysis

All experiments were conducted with four replicates (two biological and two technical), and results are presented as mean ± standard deviation (SD). All statistical analyses were performed using *R* software (version 4.4.0).

Data were grouped according to cultivation time (day of growth) and 24-epiBL concentration. To evaluate the effects of these factors, either one-way or two-way analysis of variance (ANOVA) was applied, depending on the experimental design. For parameters measured across multiple time points, a two-way ANOVA was conducted with 24-epiBL concentration and time as fixed factors. For datasets analyzed at individual time points, one-way ANOVA was used.

Prior to analysis, data were tested for normality using the Shapiro–Wilk test and for homogeneity of variances using Levene’s test, implemented in the stats and *car* packages, respectively. When the assumptions of ANOVA were not met, data were log₁₀-transformed prior to analysis. After transformation, all datasets met the assumptions of normality and homoscedasticity.

*Post hoc* comparisons were performed using Tukey’s honestly significant difference (HSD) test, implemented via the *emmeans*, *multcomp*, and *multcompView* packages. Differences were considered statistically significant at *p* < 0.05.

Statistical groupings obtained from *post hoc* analysis were visualized using letter-based annotations in bar plots generated with the *tidyverse* and *ggtext* packages. In all figures, different letters indicate statistically significant differences between treatments.

Additionally, Pearson’s correlation analysis was performed to assess relationships between the analyzed physiological and biochemical parameters under the 1 nM 24-epiBL treatment at day 8. Correlation coefficients (*r*) and corresponding *p*-values were calculated. Statistical significance was set at *p* < 0.05. Correlation matrices were visualized using the *corrplot* package.

## 3 Results

Differences among treatments and cultivation times were evaluated using one-way or two-way analysis of variance (ANOVA), depending on the dataset, followed by Tukey’s HSD *post hoc* test. Statistically significant differences (*p* < 0.05) are indicated in the figures by distinct letter groupings. Overall, *Chlamydomonas reinhardtii* exhibited a clear concentration- and time-dependent response to 24-epiBL, reflected in coordinated changes in growth dynamics, biochemical composition, pigment content, and fatty acid profiles.

### 3.1 Effects of 24-epibrassinolide on cell number of *Chlamydomonas reinhardtii*

**Figure 1:**
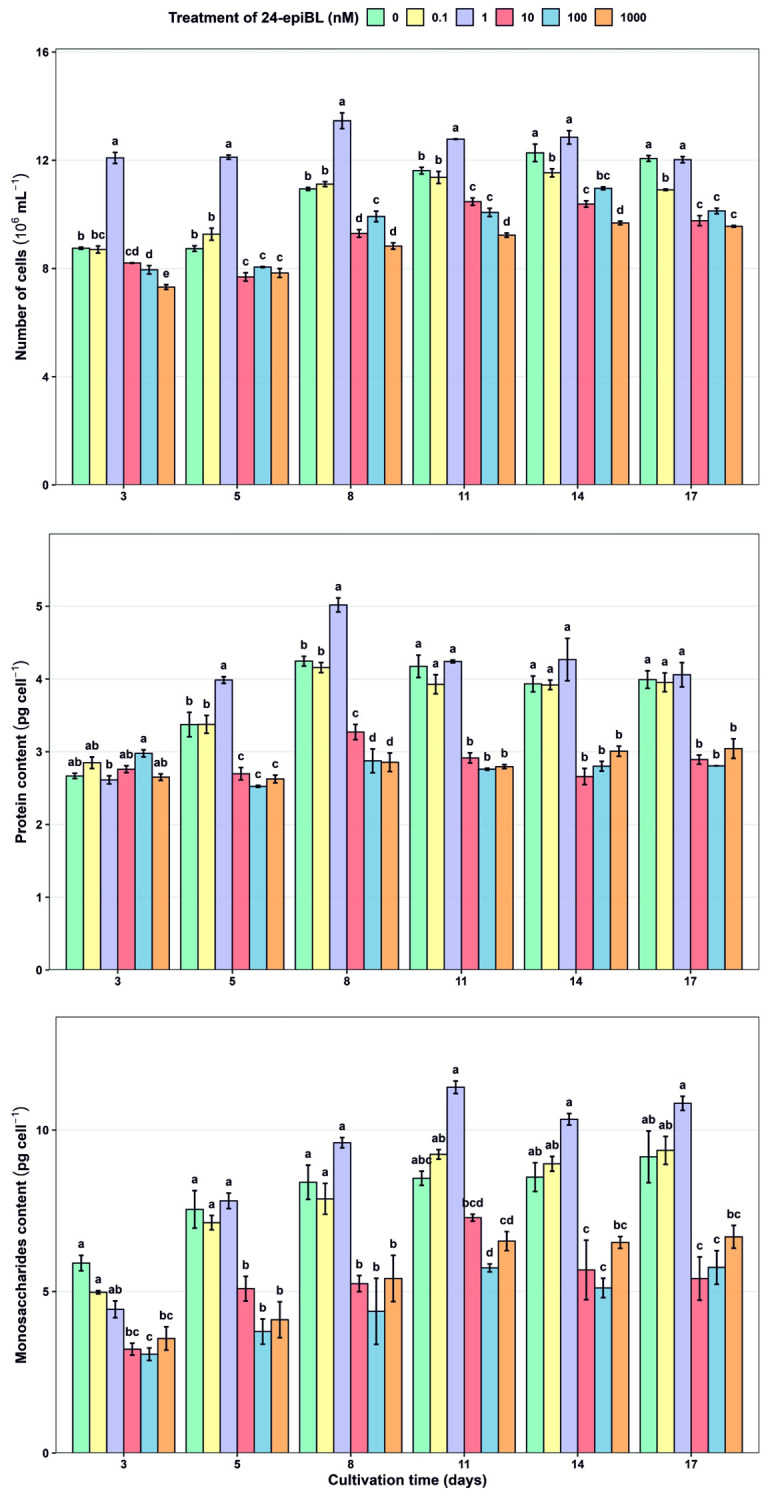
Time-dependent changes in cell density, protein and monosaccharide content in *C. reinhardtii* cultured under different concentrations of 24-epiBL. Values represent mean ± SD of 4 replicates. Different letters indicate statistically significant differences among treatments within each time point (*p* < 0.05; Tukey’s HSD test).

Cell density increased progressively in all treatments over the 17-day cultivation period (**Figure 1**). In control cultures, cell number reached a maximum on day 14, followed by a slight decline on day 17. Low concentrations of 24-epiBL (0.1 and 1 nM) stimulated cell proliferation relative to the control. The most pronounced effect was observed at 1 nM, where cell density was consistently higher than in all other treatments across the majority of sampling points. In this concentration cultures reached a maximum cell density of 13.46 ± 0.58 x 10^6^ cells/mL at day 8, which corresponds to a 23.03% increase compared to the untreated control. Notably, the divergence between the optimal (1 nM) and inhibitory treatments became apparent from day 5 onward and increased over time. Maximum cell densities in the 1 nM treatment were recorded between days 11 and 14, indicating a sustained stimulatory effect rather than a transient response. The 0.1 nM treatment also enhanced growth, although the magnitude of the effect was smaller and less consistent. In contrast, higher concentrations (10–1000 nM) reduced cell proliferation. Cultures treated with 10 nM showed moderate inhibition at later stages, whereas 100 and 1000 nM resulted in consistently lower cell densities throughout the experiment.

These results indicate a narrow optimal concentration range for 24-epiBL action, centered around 1 nM.

### 3.2 Effects of 24-epibrassinolide on protein content in *Chlamydomonas reinhardtii*

Protein content exhibited a concentration-dependent response to 24-epiBL (**Figure 1**). In control cultures, protein levels increased from day 0 to day 8, followed by stabilization or a slight decline during later cultivation stages. Low concentrations of 24-epiBL (0.1 and 1 nM) enhanced protein accumulation, with the strongest effect observed at 1 nM. At this concentration, protein content reached peak values at day 8 which got to 5.02 ± 0.19 pg/cell and that corresponds to 18.18% increase compared to the control cultures. Protein content remained elevated at subsequent time points, indicating their sustained stimulation and accumulation. The 0.1 nM treatment produced a moderate increase relative to the control, although the effect was less pronounced. In contrast, higher concentrations (10–1000 nM) resulted in consistently lower protein levels compared to both the control and low-dose treatments. Temporal variation in protein content at these concentrations was limited, and peak values were not comparable to those observed at 1 nM.

These findings demonstrate that optimal 24-epiBL concentration promotes protein accumulation, whereas higher concentrations suppress this response.

### 3.3 Effects of 24-epibrassinolide on monosaccharide content in *Chlamydomonas reinhardtii*

Monosaccharide content showed a pattern broadly consistent with growth dynamics and protein accumulation (**Figure 1**). In control cultures, monosaccharide levels increased steadily throughout the cultivation period, reaching maximum values during the late stages (days 14–17). Low concentrations of 24-epiBL (0.1 and 1 nM) promoted monosaccharide accumulation, with the strongest effect observed at 1 nM. At this concentration, monosaccharide content reached the highest levels among all treatments, particularly between days 11 and 14. The highest content of 11^th^ day in 1nM 24-epiBL cultures was 11.32 ± 0.39 pg/cell which corresponds to 33.15% growth comparing to untreated control. The temporal pattern of monosaccharide accumulation closely paralleled changes in cell density, indicating coordinated regulation of carbon accumulation and growth. The 0.1 nM treatment also increased monosaccharide levels relative to the control, although the effect was less pronounced. In contrast, higher concentrations (10–1000 nM) resulted in reduced monosaccharide accumulation. Although levels increased gradually over time, they remained consistently lower than in low-dose treatments.

These results indicate that 24-epiBL modulates carbohydrate accumulation in a concentration-dependent manner.

### 3.4 Effects of 24-epibrassinolide on pigment content in *Chlamydomonas reinhardtii*

**Figure 2:**
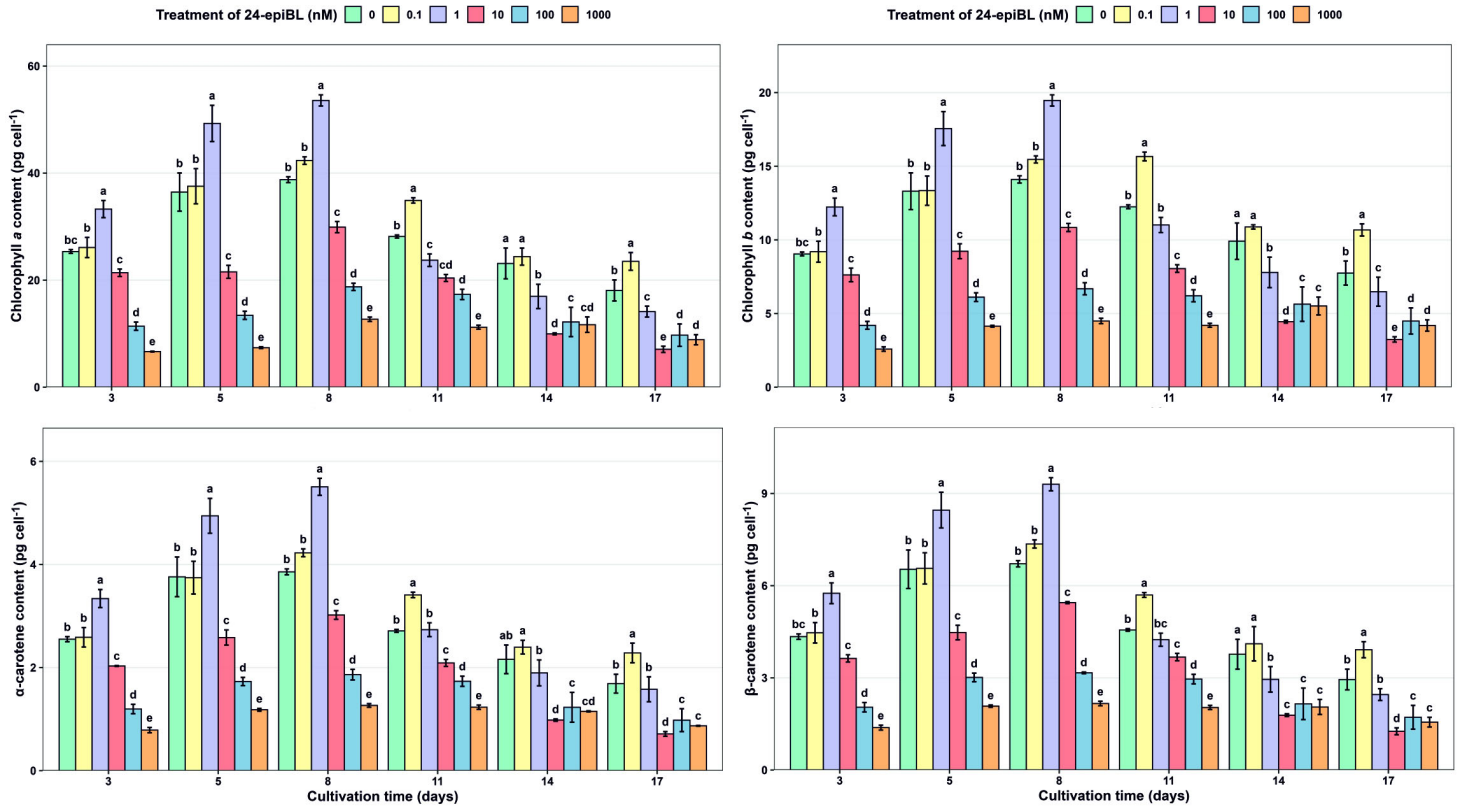
Chlorophyll and carotenoid content of *C. reinhardtii* during 17 days of cultivation under different 24-epiBL concentrations. Values represent mean ± SD of 4 biological replicates. Different letters indicate statistically significant differences among treatments within each time point (*p* < 0.05; Tukey’s HSD test).

**Figure 3:**
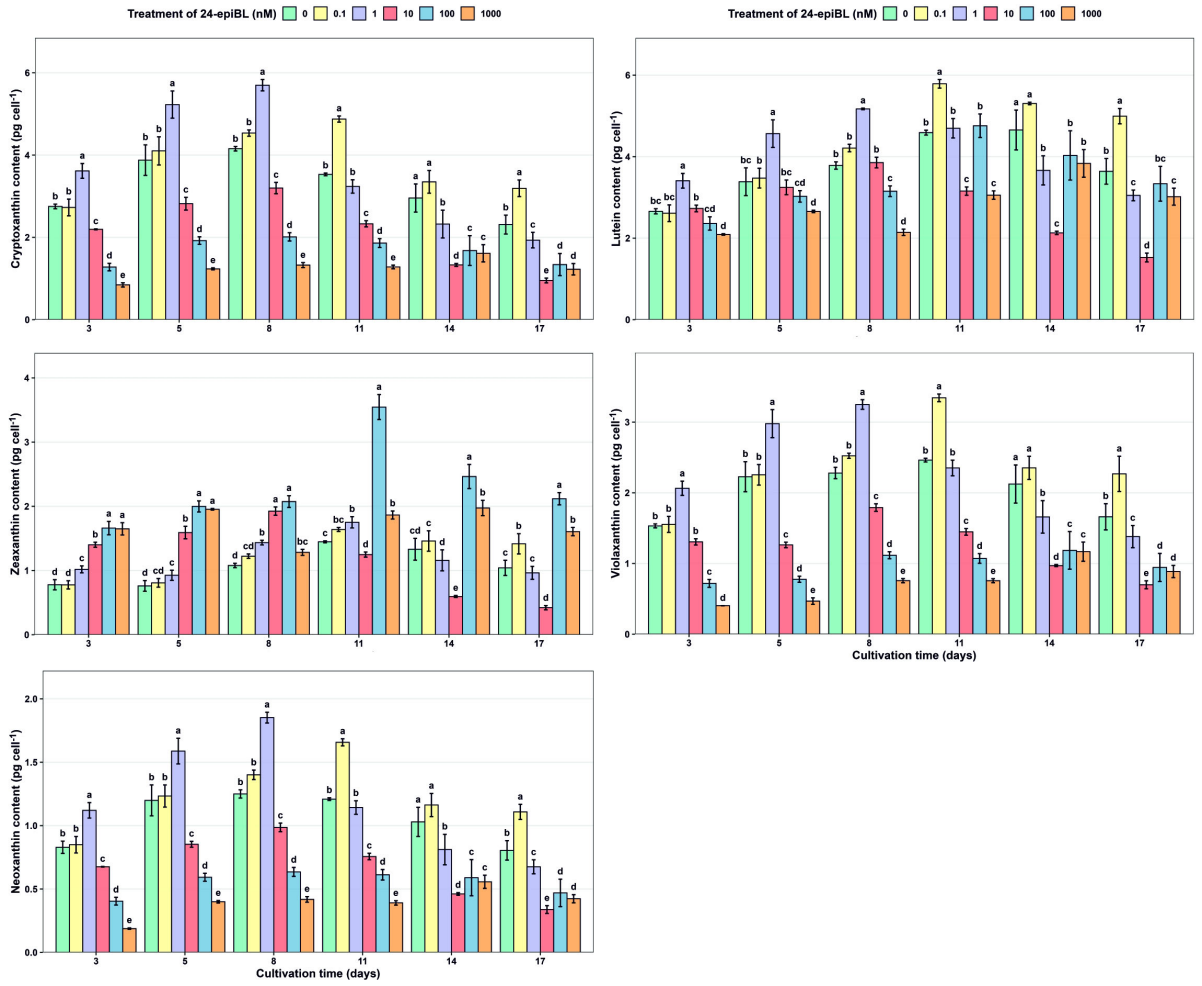
Xanthophylls content of *C. reinhardtii* during 17 days of cultivation under different 24-epiBL concentrations. Values represent mean ± SD of 4 replicates. Different letters indicate statistically significant differences among treatments within each time point (*p* < 0.05; Tukey’s HSD test).

Photosynthetic pigment content exhibited clear concentration- and time-dependent changes (**Figures 2**, **3**). In all treatments, pigment levels increased during early cultivation stages, reaching maximum values between days 5 and 8, followed by stabilization or gradual decline.

The highest pigment levels were consistently observed at 1 nM 24-epiBL. At this concentration, chlorophyll *a*, chlorophyll *b*, and major carotenoids (including lutein, β-carotene, neoxanthin, violaxanthin, and cryptoxanthin) reached the highest values among all treatments. Both chlorophylls altogether reached 73.05 ± 1.35 pg/cell which corresponds to 38.15% increase in comparison to control. However since day 11^th^, greatest amount of chlorophylls were noticed in 0.1nM 24-epiBL cultures with 50.56 ± 0.79 pg/cell on that day, which resembles 37.04% increase against control cultures. Very similar trend was observed in case of carotenoids. The highest content of total xantophylls in the whole experiment occurred also in 1nM 24-epiBL cultures on day 8^th^ with mean value of 27.51 ± 0.45 pg/cell which is 38.55% higher than in control, but in later days biggest content was present in 0.1nM 24-epiBL cultures. Although it did not reach as high content of those pigments in total. Elevated pigment levels were maintained throughout cultivation, although a gradual decline was observed after the exponential growth phase. In contrast, higher concentrations (10–1000 nM) resulted in reduced accumulation of most pigments. However, zeaxanthin exhibited a distinct pattern, with increased accumulation at elevated 24-epiBL concentrations, particularly between days 8 and 11. This divergence from other carotenoids indicates a shift in pigment composition under higher hormone concentrations.

Overall, the data demonstrate that 24-epiBL influences pigment accumulation in a concentration-dependent manner, with coordinated changes across pigment groups.

### 3.5 Effects of 24-epibrassinolide on fatty acid content in *Chlamydomonas reinhardtii*

**Figure 4:**
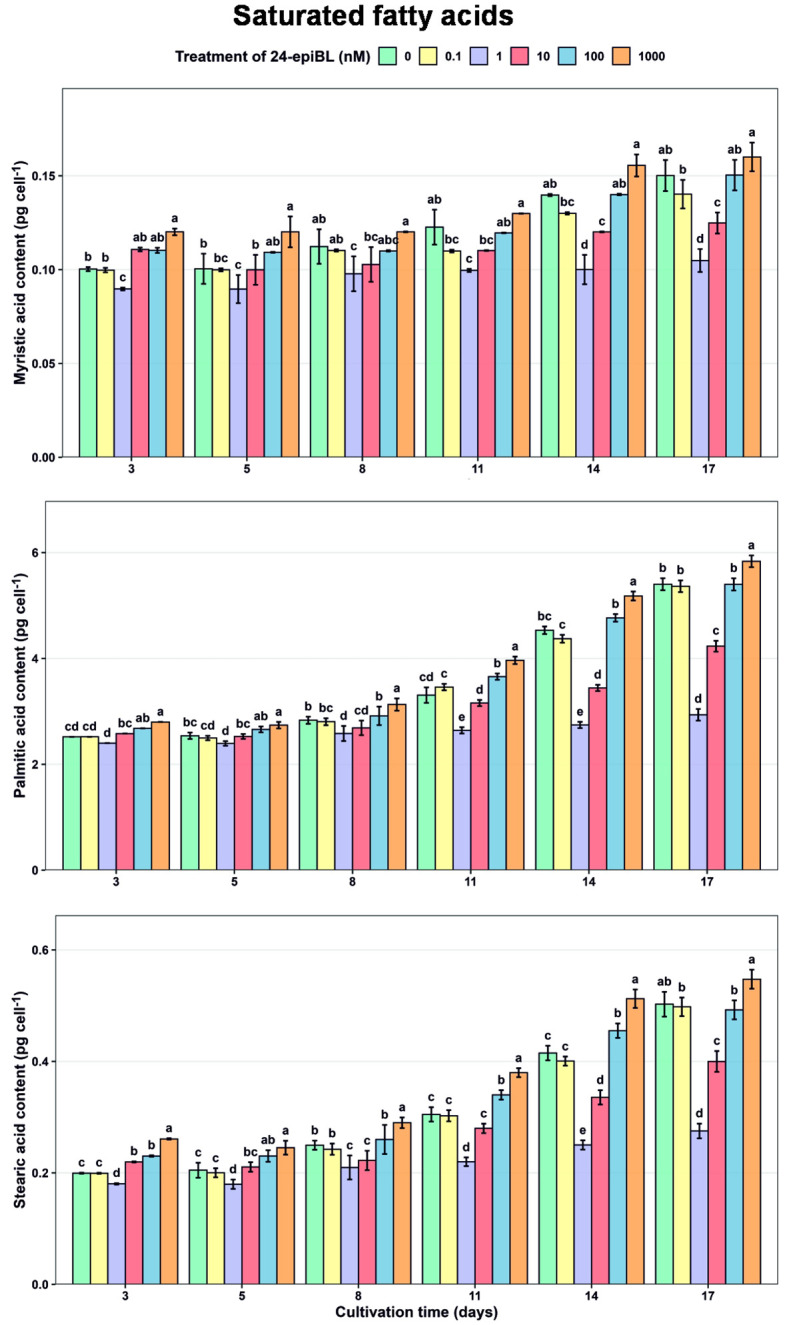
Saturated fatty acid composition of *C. reinhardtii* under different 24-epiBL concentrations. Values represent mean ± SD of 4 replicates. Different letters indicate statistically significant differences among treatments within each time point (*p* < 0.05; Tukey’s HSD test).

**Figure 5:**
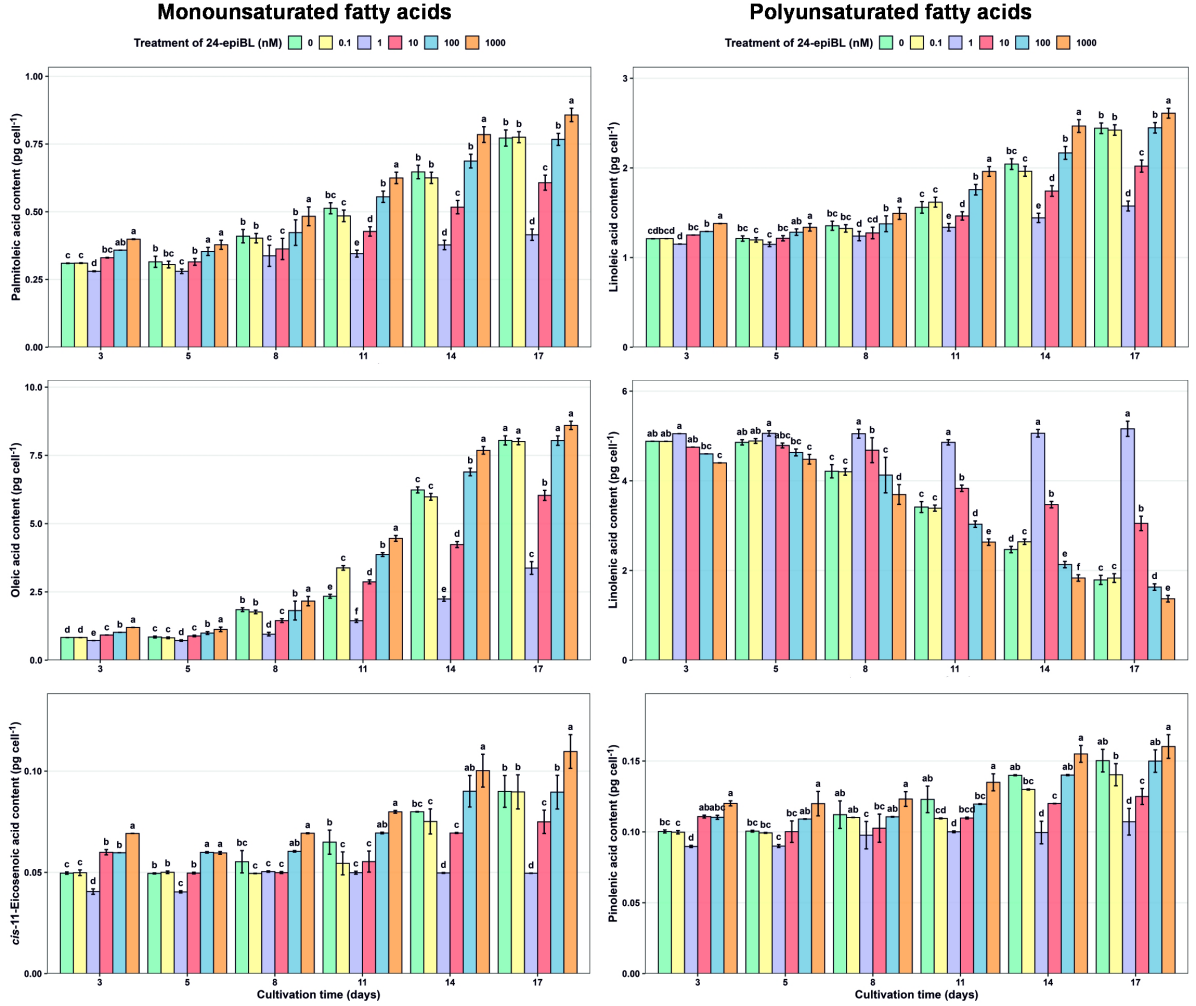
Monounsaturated and polyunsaturated fatty acid composition of *C. reinhardtii* under different 24-epiBL concentrations. Values represent mean ± SD of 4 replicates. Different letters indicate statistically significant differences among treatments within each time point (*p* < 0.05; Tukey’s HSD test).

Fatty acid composition was significantly influenced by 24-epiBL concentration and cultivation time (**Figures 4**, **5**). Clear differences were observed among saturated (SFAs), monounsaturated (MUFAs), and polyunsaturated fatty acids (PUFAs).

In general, the levels of several fatty acids, including myristic (C14:0), palmitic (C16:0), palmitoleic (C16:1), stearic (C18:0), oleic (C18:1n9c), and linoleic acid (C18:2n6c), increased over time in all treatments. However, the magnitude of these increases was dependent on 24-epiBL concentration, with more pronounced accumulation observed at higher concentrations (100–1000 nM). In contrast, linolenic acid (C18:3n3) displayed a decreasing trend over time, particularly at elevated 24-epiBL concentrations. At 1 nM, its level remained relatively stable throughout cultivation, distinguishing this treatment from both control and high-dose conditions. On the 17^th^ day its content reach value of 5.16 ± 0.17 pg/cell which corresponds to 188.29% increase comparing to control cultures.

Low concentrations of 24-epiBL, particularly 1 nM, were associated with relatively higher proportions of unsaturated fatty acids during the exponential growth phase. In contrast, higher concentrations (10–1000 nM) promoted a shift toward increased proportions of saturated fatty acids, especially during later cultivation stages (days 14–17).

These results indicate that 24-epiBL modulates fatty acid composition in a concentration-dependent manner, resulting in distinct lipid profiles under optimal and inhibitory conditions.

### 3.6 Correlation analysis

**Figure 6:**
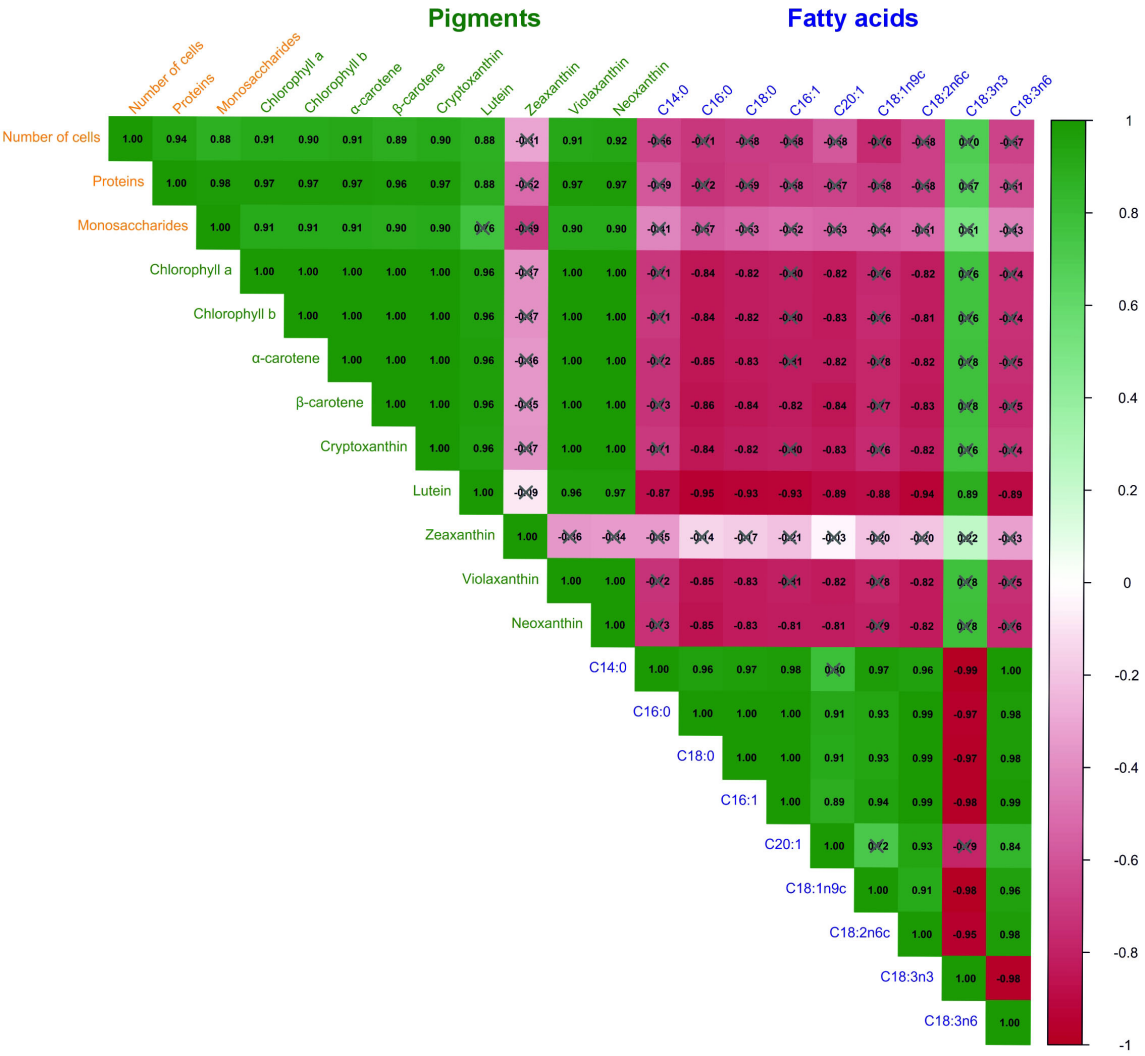
Pearson correlation heatmap of cell number, protein content, monosaccharide content, pigment levels, and fatty acid composition in *C. reinhardtii* treated with 1 nM 24-epiBL at day 8 of cultivation. Color intensity represents the strength and direction of the correlation coefficient (*r*), with green indicating positive correlations, red indicating negative correlations, and white indicating no correlation. Crossed-out cells denote statistically non-significant correlations (*p* ≥ 0.05).

Correlation analysis of all measured parameters at day 8, corresponding to the peak of cell growth, revealed a highly structured pattern of relationships among physiological and biochemical traits (**Figure 6**).

Cell number, protein content, and monosaccharide levels were strongly and positively correlated with each other, forming a consistent cluster of growth-related variables. These parameters also showed strong positive correlations with chlorophylls and most carotenoids, indicating a close association between biomass accumulation, primary metabolism, and pigment content.

In contrast, most fatty acids displayed predominantly negative correlations with growth- and pigment-related parameters. This pattern was particularly evident for saturated and monounsaturated fatty acids, which were negatively associated with chlorophylls, carotenoids, and primary metabolites. At the same time, these fatty acids exhibited strong positive correlations among themselves, indicating coordinated variation within the lipid fraction.

Linolenic acid (C18:3n3) exhibited a distinct correlation pattern compared to other fatty acids. It showed negative correlations with several lipid components and largely weak or non-significant relationships with growth and pigment variables, indicating a differentiated behavior within the fatty acid group.

Zeaxanthin also displayed a distinct profile, with predominantly weak or non-significant correlations with most other variables, in contrast to the strong positive relationships observed among other carotenoids.

Overall, the correlation matrix revealed two clearly distinguishable groups of variables: (i) growth- and photosynthesis-associated parameters, including cell number, proteins, monosaccharides, chlorophylls, and most carotenoids, and (ii) lipid-related parameters, which followed a contrasting correlation pattern. These relationships should be interpreted as associative rather than causal.

## 4 Discussion

The present study demonstrates that exogenous 24-epiBL exerts a strong concentration-dependent influence on the growth and metabolic profile of *C. reinhardtii*. Such biphasic responses are widely reported for BRs in higher plants and have also been documented in algae, where low concentrations stimulate growth and metabolism, whereas higher doses inhibit these processes (Bajguz and Czerpak, 1996; Bajguz and Hayat, 2009; Bajguz, 2010; Talarek-Karwel et al., 2018; Du et al., 2020). In the present study, the overall response pattern was consistent with hormesis, and 1 nM 24-epiBL emerged as the most effective concentration for enhancing biomass-related traits. 24-epibrassinolide is characterized by relatively high stability in aqueous solutions under standard physiological conditions. Although its long-term abiotic degradation was not explicitly monitored, in algal physiology, phytohormone treatments typically act as an initial signaling trigger that reprograms cellular metabolism early in the growth phase (Bajguz, 2010; Bajguz and Piotrowska-Niczyporuk, 2014). Therefore, even if partial abiotic degradation occurred during the later stages of cultivation, the initial perception of 24-epiBL was sufficient to induce a cascading, long-term physiological shift, as evidenced by the robust and dose-dependent metabolic changes observed throughout the experiment.

**Figure 7:**
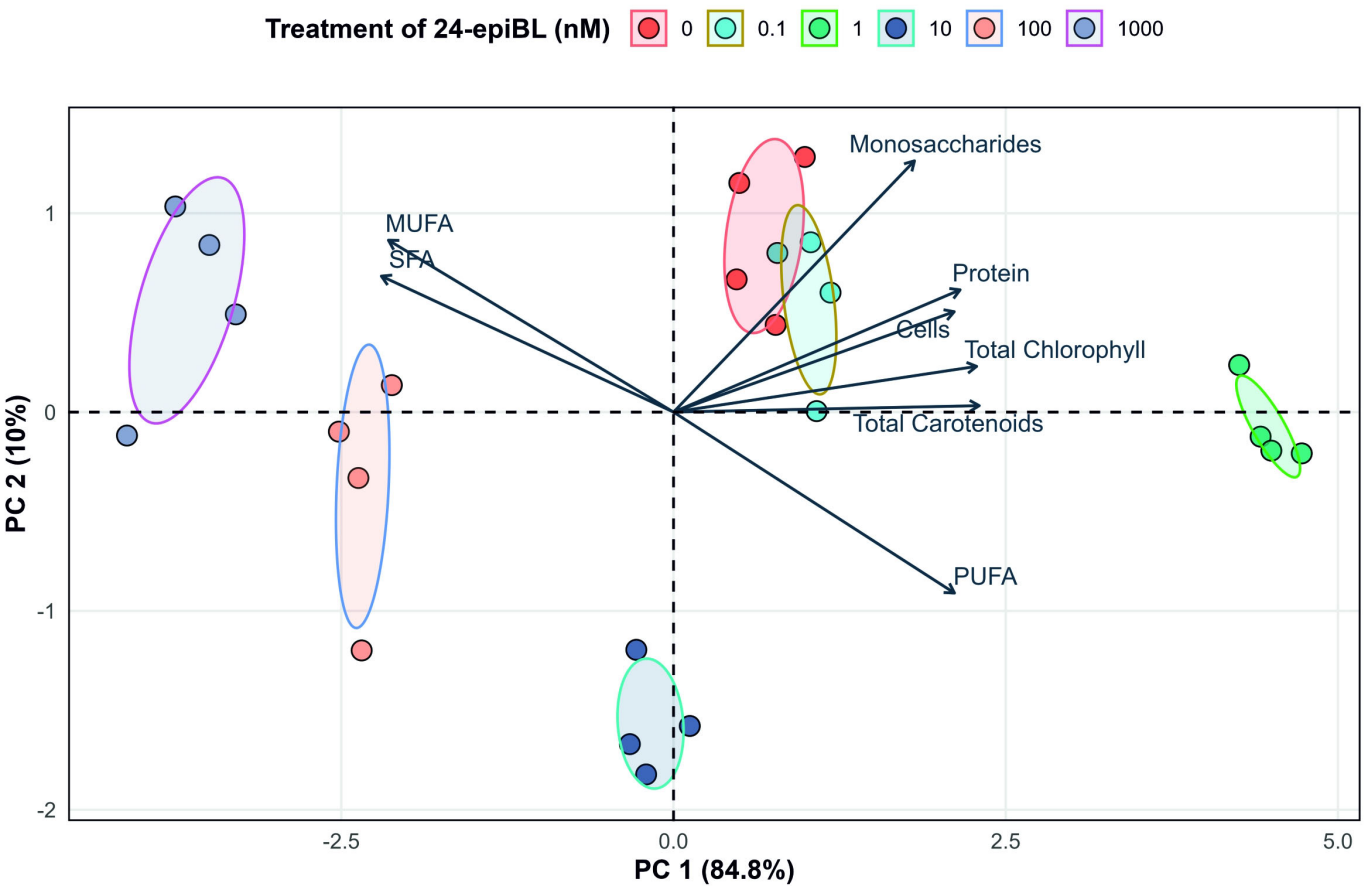
Principal component analysis (PCA) biplot representing the physiological state of *C. reinhardtii* on day 8 of exposure to varying concentrations of 24-epiBL. Points represent individual replicates for each treatment group (0–1000 nM). Colored shaded areas delineate the 95% confidence ellipses for each concentration. Vectors (arrows) indicate the contribution and correlation of the analyzed physiological and biochemical parameters with the first two principal components (PC1 and PC2).

The PCA biplot (**Figure 7**) supports multivariate separation among treatments, consistent with coordinated physiological responses. Specifically, the 1 nM 24-epiBL treatment clustered closely with enhanced biomass parameters, whereas higher concentrations shifted the overall physiological state away from proliferation. This supports the view that BRs act within a relatively narrow effective concentration range, with supra-optimal doses causing a coordinated shift in the physiological state away from proliferation.

In higher plants, BRs are known to enhance cell cycle activity and biosynthetic processes (Fridman and Savaldi-Goldstein, 2013), which is consistent with the increased protein content observed in algal cells at 1 nM 24-epiBL. Importantly, the growth-promoting response at this concentration was sustained across multiple sampling points, indicating a stable physiological effect rather than a transient fluctuation. Greatest cell number in 1 nM concentration was reached about 8^th^ day. Similar amounts of biomass in control cultures were reached much later, on 11-14^th^ day. Such knowledge might be very useful for industrial/commercial purposes due to immense potential of *C. reinhardtti* in various fields.

In contrast, reduced cell density and protein content at higher 24-epiBL concentrations likely reflect a broader reallocation of cellular resources under conditions where growth is constrained. Although gene expression and enzymatic activities were not measured in this study, supra-optimal BR signaling has been reported to disturb metabolic balance in plant systems (Guo et al., 2013), and a similar shift may contribute to the reduced biosynthetic output observed here.

The protein data reinforce this interpretation. At 1 nM 24-epiBL, protein content per cell was clearly elevated, particularly during the mid-cultivation phase, when growth stimulation was most pronounced. As highlighted by the PCA, this coordinated increase in cell number and cellular protein content strongly suggests that low-dose 24-epiBL does not simply accelerate cell division at the expense of biomass quality, but rather supports a globally anabolic physiological state (**Figure 7**). In microalgae, increased protein accumulation may reflect enhanced nitrogen assimilation, elevated translational activity, or improved maintenance of photosynthetically derived carbon skeletons required for amino acid biosynthesis. Although the present study does not resolve which of these processes predominates, the observed clustering of growth parameters confirms that 24-epiBL at optimal concentration promotes biosynthetic competence rather than merely increasing cell number (Bajguz and Piotrowska-Niczyporuk, 2014).

By contrast, the reduced protein content observed at higher BR concentrations indicates that supra-optimal hormone exposure constrains anabolic metabolism. This may result from reduced photosynthetic efficiency, altered nutrient allocation, or activation of protective processes that divert resources away from growth-related pathways. As no molecular markers of stress or nitrogen metabolism were assessed, these explanations remain hypothetical; however, the observed pattern is consistent with a shift from growth-oriented metabolism toward a maintenance-oriented physiological state under non-optimal hormonal conditions (Jantapo et al., 2021; Erofeeva, 2022).

A similarly coordinated pattern was observed for monosaccharides. Their accumulation was highest at 1 nM 24-epiBL and, as shown by the PCA vectors, broadly paralleled both cell density and pigment content, suggesting that BR treatment at this concentration improves the balance between carbon acquisition and utilization. In mixotrophic algal systems, elevated monosaccharide content may reflect enhanced photosynthetic carbon fixation, altered carbon partitioning, or a combination of both processes.

The temporal dynamics of monosaccharide accumulation are particularly noteworthy. In the optimal treatment (1nM), monosaccharide levels remained elevated during periods when both pigment content and cell density were high, suggesting that BRs may enhance overall metabolic capacity rather than simply delaying senescence. In contrast, monosaccharide accumulation was attenuated at higher BR concentrations. This may potentially indicate reduced carbon assimilation, increased consumption of soluble sugars in maintenance or protective processes, or disruption of carbon allocation into growth-supporting pathways.

Monosaccharide content closely followed protein dynamics, indicating coordinated regulation of carbon and nitrogen metabolism across the dose range. BRs have been reported to enhance photosynthesis-related traits in plants, including chlorophyll accumulation and photosystem performance (Siddiqui et al., 2018), as well as to protect the photosynthetic apparatus under stress conditions (Li et al., 2015). Consistent with this, the increased monosaccharide content at 1 nM 24-epiBL coincided with elevated pigment levels and enhanced growth, suggesting improved carbon gain and/or allocation. At higher concentrations, reduced monosaccharide levels may point to altered carbon metabolism under conditions where growth is inhibited. Whether this reflects decreased assimilation, altered partitioning, or increased metabolic consumption cannot be resolved from the present dataset and requires further investigation using direct physiological measurements.

Pigment data provide additional insight into how 24-epiBL modulates cellular physiology across the dose range. At 1 nM 24-epiBL, chlorophyll *a*, chlorophyll *b*, and major carotenoids (including lutein, β-carotene, neoxanthin, violaxanthin, and cryptoxanthin) reached the highest levels, consistent with enhanced accumulation of light-harvesting and accessory pigments under growth-promoting conditions. Similar trends have been reported in other green microalgae, such as *Chlorella vulgaris* (Bajguz and Piotrowska-Niczyporuk, 2013, Bajguz and Piotrowska-Niczyporuk, 2014). On the other hand in the cultures with 0.1 nM 24-epiBL we witnessed that in later days (11-17^th^ day) of growth, *C. reinhardtii* outperformed other cultures in terms of producing and accumulating most of the pigments and fatty acids. The fact of cultures entering stationary phase with 0.1 nM of hormone, increased their content in analyzed species. Such knowledge might also be useful in commercial culturing of this species, depending on expected outcome.

The simultaneous increase in chlorophylls and carotenoids, together with elevated cell density and monosaccharide content, indicates that 1 nM 24-epiBL significantly modulates the photosynthetic pigment profile. While these coordinate changes suggest that BR supplementation supports the accumulation of light-harvesting components compatible with active growth (Polle et al., 2001), pigment content alone does not directly equate to functional efficiency. As direct measurements of photosynthetic activity (e.g., oxygen evolution) were not performed in this study, the exact functional effect on overall photosynthetic capacity remains to be fully elucidated. Importantly, the stimulation was not restricted to a single pigment class, indicating a broad effect on plastid-associated metabolism. This provides a physiological basis for the observed increases in protein and carbohydrate content, linking pigment dynamics with enhanced anabolic output.

In contrast, cultures treated with higher 24-epiBL concentrations exhibited reduced levels of most photosynthetic pigments. A notable exception was zeaxanthin, which accumulated more strongly under elevated BR concentrations. Zeaxanthin is closely associated with photoprotective energy dissipation mechanisms and the xanthophyll cycle (Frank et al., 2000; Demmig-Adams et al., 2025). Its relative enrichment under supra-optimal BR conditions suggests a shift from a growth-oriented pigment profile toward a protection-oriented configuration. Although direct measurements of photoprotection or oxidative stress were not performed, the coexistence of reduced chlorophyll content, lower growth, and increased zeaxanthin supports the interpretation that cellular energy dissipation processes may become more prominent under these conditions.

The lipid data further support the existence of two contrasting BR response states: a low-dose, productivity-associated state and a high-dose, maintenance-oriented state. At 1 nM, the fatty acid profile was characterized by a relatively higher abundance of selected PUFAs, accompanied by lower proportions of SFAs and MUFAs. Similar patterns have been reported in higher plants treated with BRs and are often associated with enhanced membrane fluidity and metabolic activity (Fedina et al., 2017; Chen et al., 2023). Although membrane properties and desaturase activities were not directly assessed, BRs are known to influence lipid metabolism, including pathways controlling fatty acid desaturation (Shi et al., 2023), and such mechanisms may contribute to the observed patterns.

In contrast, higher 24-epiBL concentrations were associated with increased proportions of saturated fatty acids, particularly during later cultivation stages. Such shifts have been linked to altered membrane properties and reduced metabolic efficiency in aging or stressed algal cultures (Heredia-Martínez et al., 2018). This divergence from the low-dose profile further supports the view that supra-optimal BR concentrations induce a distinct physiological state that does not favor lipid configurations associated with active growth (Jiang et al., 2013). Notably, linolenic acid (C18:3n3) levels were markedly higher in cultures treated with 1 nM 24-epiBL and are closely associated with the maintenance and protection of photosystem II (Murakami et al., 2000; Szilágyi, 2007). This pattern is consistent with the observed increases in monosaccharide content and photosynthetic pigments, collectively suggesting improved photosynthetic efficiency under optimal BR conditions.

In contrast, oleic acid (C18:1n9c), which serves as a precursor for linoleic and linolenic acids, was present at relatively lower levels in the 1 nM treatment. This may reflect enhanced metabolic flux toward PUFA synthesis, resulting from more active lipid metabolism (Li-Beisson et al., 2015). At higher 24-epiBL concentrations, fatty acid profiles differed markedly, with a stronger contribution of saturated fatty acids, particularly during later cultivation stages. This shift may be associated with altered photosynthetic performance and lipid remodeling processes under non-optimal conditions (Hu et al., 2016).

Taken together, the results indicate that 24-epiBL modulates growth, metabolism, pigment composition, and lipid profiles in *C. reinhardtii* in a dose- and time-dependent manner. Low nanomolar concentrations promote traits associated with productivity and metabolic efficiency, whereas higher concentrations shift the cellular state toward reduced growth and altered metabolic balance. This trade-off is consistent with a hormetic model of BR action and highlights the importance of precise dose control.

One of the key strengths of the dataset is the consistency among independent physiological parameters. The optimal BR treatment simultaneously enhanced cell density, protein and monosaccharide content, pigment levels, and fatty acid composition, indicating coordinated regulation rather than isolated biochemical changes. This supports a systems-level interpretation in which low-dose 24-epiBL improves overall metabolic integration in *C. reinhardtii*. The temporal dimension of the response is also noteworthy. The effects of BR treatment varied across cultivation stages, with the strongest stimulation often observed during the mid-growth phase. This suggests that BR action is influenced by the physiological state of the culture, implying that both concentration and timing of application may be critical factors in practical applications.

Finally, it should be emphasized that the present study focuses on physiological and biochemical responses and does not address underlying molecular mechanisms. Future research integrating transcriptomic, metabolomic, and physiological approaches, including measurements of photosynthetic performance and stress-related parameters, will be necessary to fully elucidate how BRs regulate algal metabolism and stress responses.

## 5 Conclusions

This study demonstrates that 24-epiBL exerts a clear concentration-dependent effect on the growth and metabolic characteristics of *C. reinhardtii*. Among the tested concentrations, 1 nM 24-epiBL was identified as optimal, significantly increasing cell density as well as protein and monosaccharide accumulation. This concentration was also associated with a shift in lipid composition toward a higher proportion of unsaturated fatty acids and with increased levels of photosynthetic and carotenoid pigments, which may indicate enhanced photosynthetic capacity and metabolic performance. Further measurements like oxygen evolution or chlorophyll fluorescence needs to be performed to confirm that statement.

In contrast, higher concentrations of 24-epiBL reduced growth and altered biochemical profiles, consistent with a shift toward a less favorable physiological state. The lowest concentration tested (0.1 nM) elicited only minor or inconsistent responses, highlighting the relatively narrow effective range of BR action in this microalgal species.

Overall, these findings support a hormetic model of BR activity in *C. reinhardtii* and extend current understanding of BR-mediated regulation beyond higher plants. Controlled application of BRs may therefore represent a promising approach for modulating growth and metabolic traits in microalgae, with potential relevance for biotechnological applications.

Further studies integrating molecular analyses, direct measurements of photosynthetic performance, and long-term cultivation experiments will be required to elucidate the mechanisms underlying BR action and to assess their applicability under production-relevant conditions.

## Data Availability

Publicly available datasets were analyzed in this study. This data can be found here: The datasets generated and/or analyzed during the current study are not publicly available but are available from the corresponding author upon reasonable request.
